# Supplementary material for: A Lin28 homologue reprograms differentiated cells to stem cells in the moss Physcomitrella patens
Source: Nat Commun. 2017 Jan 27;8:14242. doi: 10.1038/ncomms14242 (PMC5290140; doi:10.1038/ncomms14242)
Supplement: Supplementary Information — Supplementary Figures, Supplementary Tables and Supplementary References [file ncomms14242-s1.pdf]

Supplementary Figure 1

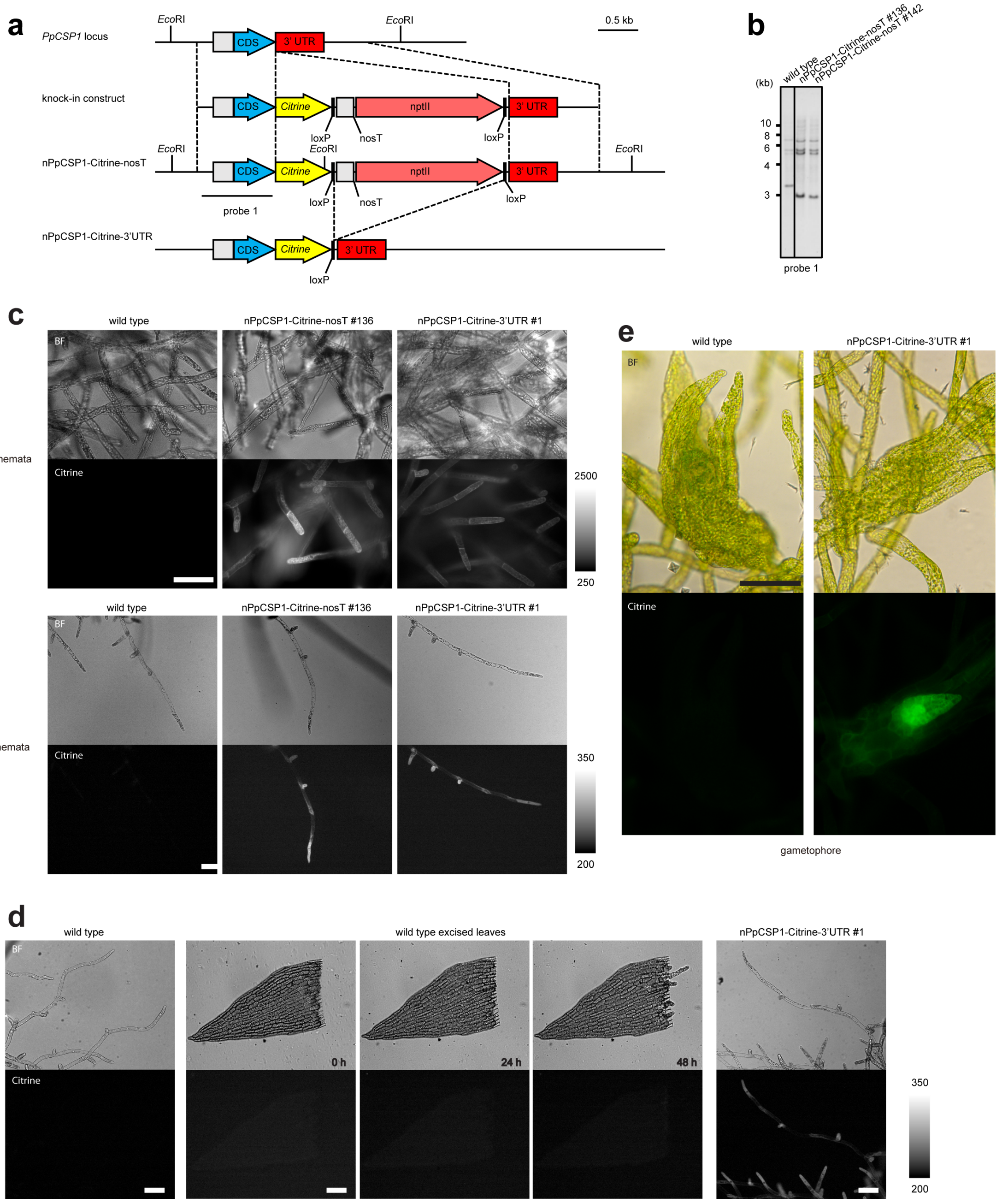

Supplementary Figure 1. Construction and observation of the nPpCSP1-Citrine-nosT and nPpCSP1-Citrine-3'UTR lines

(**a**) Schematic showing the insertion of a Citrine expression construct into the *PpCSP1* locus and removal of the nopaline synthase polyadenylation signal (nosT) [1] and neomycin

phosphotransferase II expression cassette (nptII) [2]. Blue, yellow, and pink arrows represent the *PpCSP1* coding sequence (CDS), *Citrine* CDS [3], and the nptII expression cassette, respectively. Gray and red boxes denote nosT and 3' UTR of *PpCSP1*, respectively. The probe used in (b) is indicated. The nosT and nptII expression cassette were removed from the genome by transiently introducing plasmid DNA encoding Cre enzyme to produce nPpCSP1-Citrine-3'UTR lines (bottom).

(**b**) DNA gel-blot analysis of targeted lines. Genomic DNA of wild type and nPpCSP1-Citrine-nosT (#136 and #142) lines was digested with *Eco*RI.

(**c**) Bright-field (BF) and fluorescent (Citrine) images of chloronemata and caulonemata of the wild type, nPpCSP1-Citrine-nosT #136 line, and nPpCSP1-Citrine-3'UTR #1 line. Images were taken under the same experimental conditions mentioned in Methods and PpCSP1-Citirne fluorescence was strong enough to be distinguished from autofluorescence.

(**d**) Bright-field (BF) and fluorescent (Citrine) images of excised leaves and caulonemata of the wild type as well as caulonemata of nPpCSP1-Citrine-3'UTR #1 line. Images were taken under the same experimental conditions mentioned in Methods and PpCSP1-Citirne fluorescence was strong enough to be distinguished from autofluorescence.

(**e**) Bright-field (BF) and fluorescent (Citrine) images of gametophore apices of the wild type

and nPpCSP1-Citrine-3’UTR #1 line.

Scale bars represent 100 µm in (**c**-e).

Supplementary Figure 2

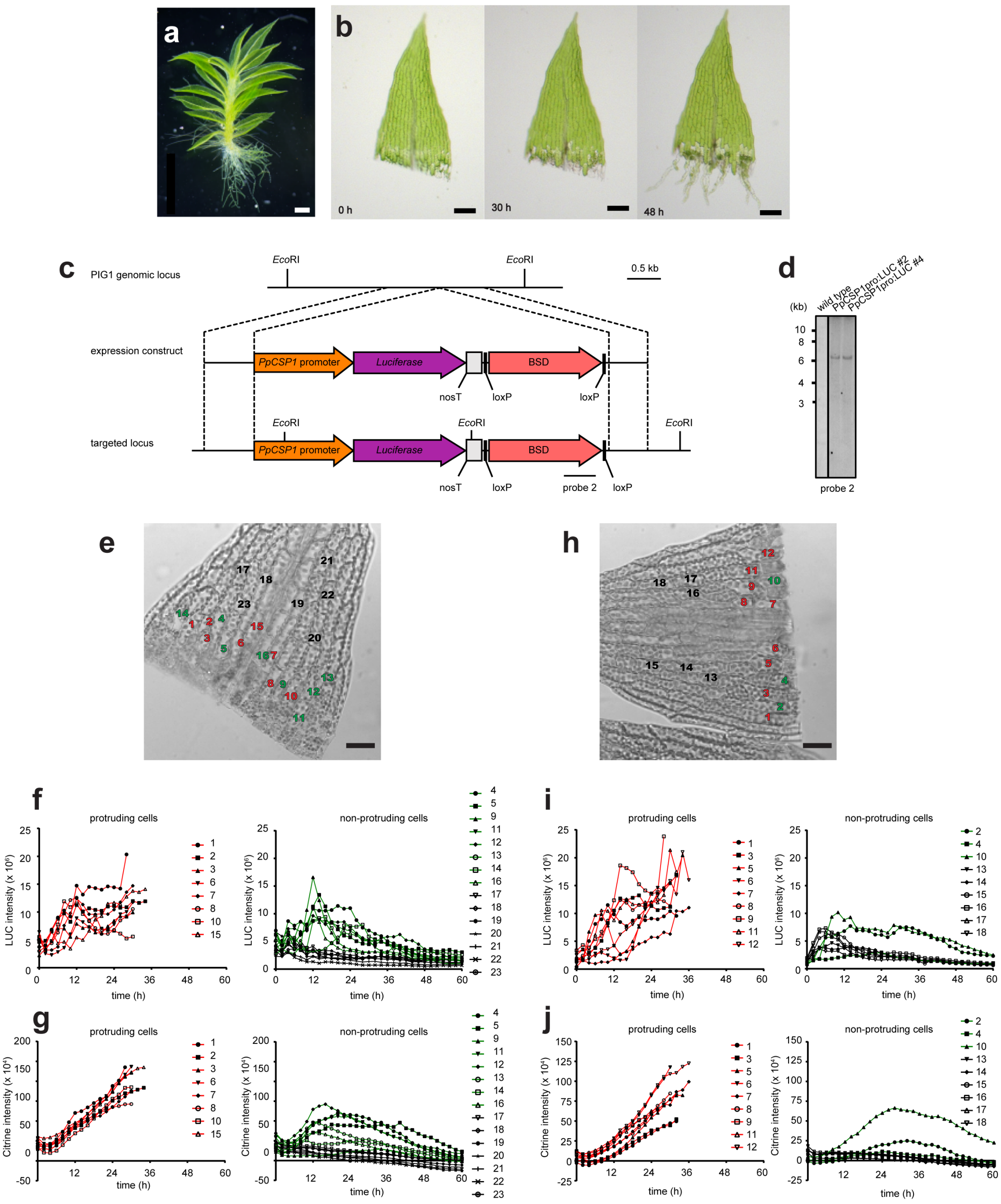

Supplementary Figure 2. Construction and quantitative analyses of LUC luminescence and Citrine fluorescence of the PpCSP1pro:LUC nPpCSP1-Citrine-3'UTR lines

(**a**) A wild-type gametophore 4 weeks after inoculation.

(**b**) A wild-type leaf at 0, 30, and 48 hours after excision.

(**c**) Schematic showing the insertion of the PpCSP1pro:LUC construct into the PIG1 locus [4,5] in nPpCSP1-Citrine-3'UTR background. Orange, purple, and pink arrows denote the *PpCSP1* promoter, the DNA fragment encoding luciferase protein, and the blasticidin S deaminase expression cassette (BSD) [1], respectively. Gray and black boxes denote the nosT and the loxP [6] sequence, respectively. The probe used in (**d**) is indicated.

(**d**) DNA gel-blot analysis of targeted lines. Genomic DNA of wild type and PpCSP1pro:LUC (#2 and #4) lines was digested with EcoRI.

(**e-j**) Replicates of quantitative analysis of *PpCSP1* promoter activity and the protein amount during the reprogramming of the PpCSP1pro:LUC nPpCSP1-Citrine-3'UTR #2 line. Cut leaves of the PpCSP1pro:LUC nPpCSP1-Citrine-3'UTR #2 line used for quantitative analysis of PpCSP1 promoter activity and the protein amount during the reprogramming. All edge cells and several non-edge cells are numbered for (**f**,**i**) and (**g**,**j**), respectively.

(**f**,**g**,**i**,**j**) The intensity of luciferase (**f**,**i**) and Citrine (**g**,**j**) signals at each cell (indicated by 1 to 23 in [**e**] and 1 to 18 in [**h**], respectively) in excised leaves. Red and green lines indicate the signal intensity in edge cells that are and are not reprogrammed into stem cells, respectively. Black lines indicate the signal intensity in non-edge cells that are not reprogrammed into stem cells.

Scale bars represent 500 μm in (**a**), 100 μm in (**b**), and 50 μm in (**e**) and (**h**).

Supplementary Figure 3

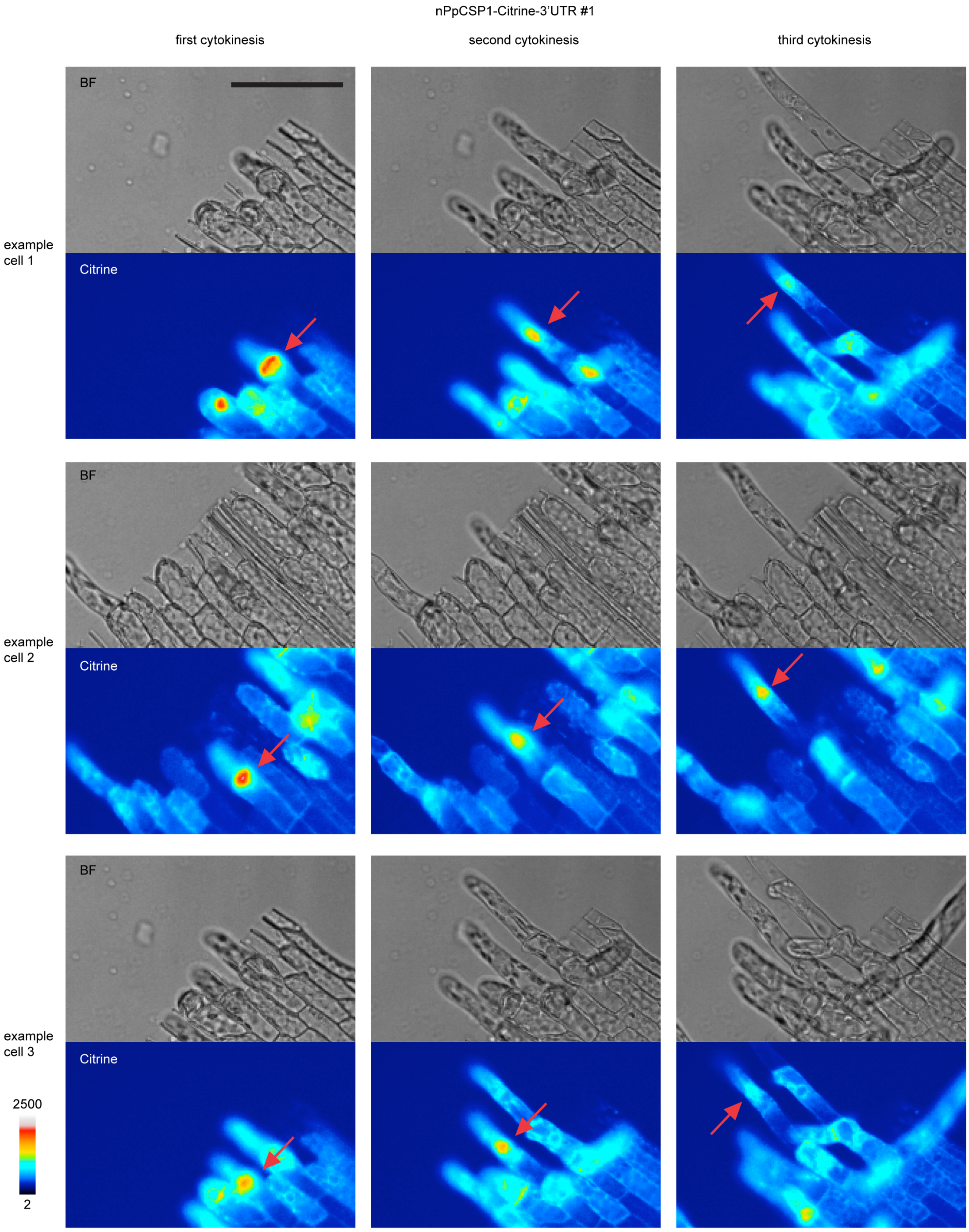

Supplementary Figure 3. PpCSP1-Citrine protein localized at the phragmoplasts in excised leaf cells of nPpCSP1-Citrine-3'UTR #1 line.

Initial three rounds of cytokinesis in chloronema apical stem cells reprogrammed from leaf cells are shown. Three chloronema apical stem cells from an excised leaf are shown. Red arrows indicate the phragmoplasts. See also Supplementary Movie 2

Scale bars represent 100 μm.

Supplementary Figure 4

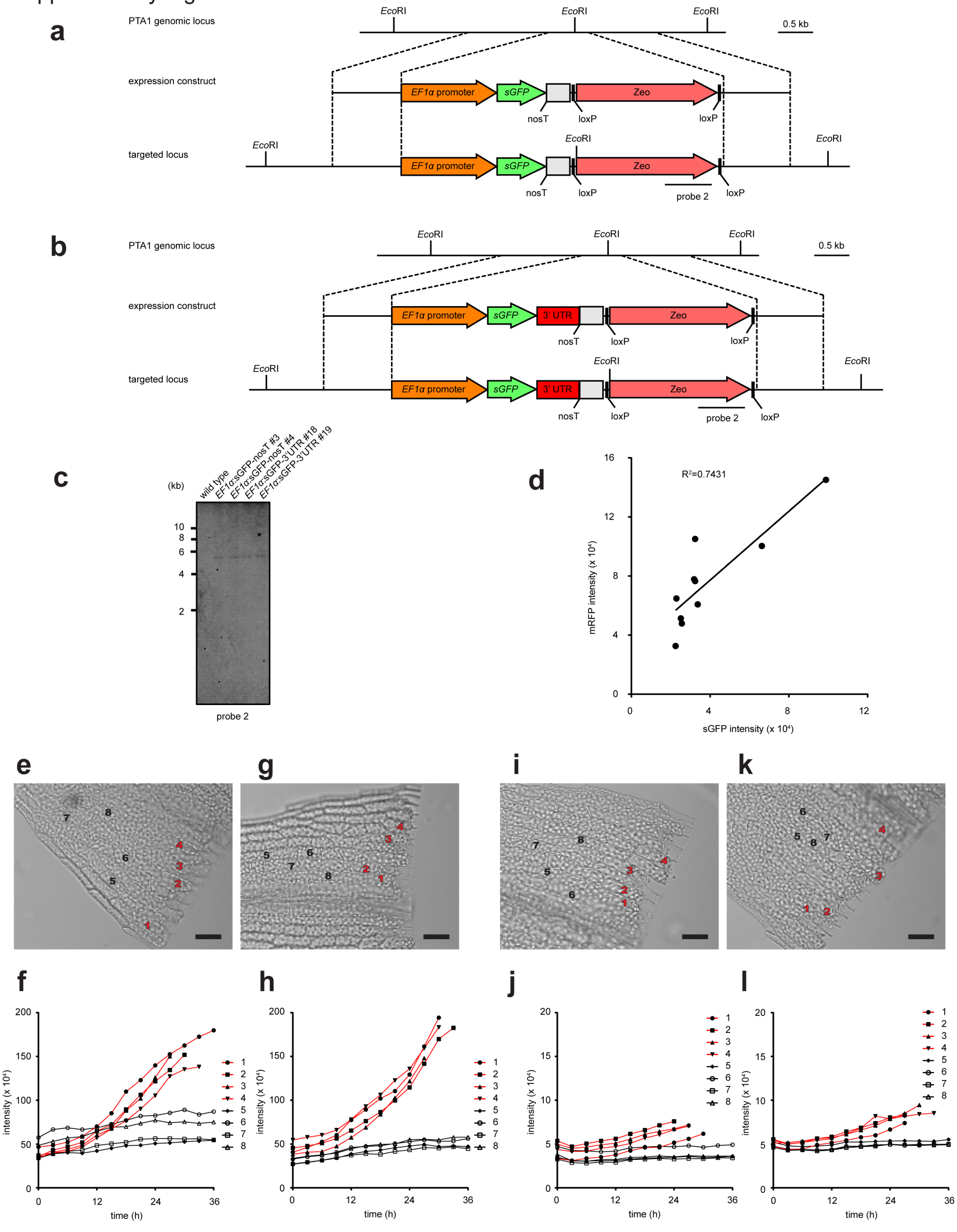

Supplementary Figure 4. Construction of the EF1αpro:sGFP-nosT and EF1αpro:sGFP-3'UTR lines

(**a**,**b**) Schematic showing the insertion of a *sGFP* reporter gene driven by the *EF1α*

promoter into the PTA1 locus with nosT (**a**) or 3' UTR of *PpCSP1* gene (**b**). Orange, green, and pink arrows indicate *EF1α* promoter [7], *sGFP* gene [8], and zeocin resistance cassette (Zeo) [9] respectively. Gray, black, and red boxes denote nosT, loxP, and *PpCSP1* 3' UTR, respectively. The probe used in (**c**) is indicated.

(**c**) DNA gel-blot analysis of targeted lines. Genomic DNA of wild type, EF1αpro:sGFP-nosT (#3 and #4), and EF1αpro:sGFP-3'UTR (#18 and #19) lines was digested with *Eco*RI.

(**d**) Linear correlation of the sGFP and mRFP [10] signals in the cells transformed with both constructs. X- and Y-axes indicate the fluorescence intensity of sGFP and mRFP, respectively.

(**e**-**l**) Replicates of quantitative analysis of sGFP signals of EF1αpro:sGFP-nosT #3 (**e**-**h**) and EF1αpro:sGFP-3'UTR #18 (**i**-**k**) lines. Cut leaves of EF1αpro:sGFP-nosT #3 (**e,g**) and EF1αpro:sGFP-3'UTR #18 (**i**,**k**) lines for quantitative analysis of sGFP signals. Several edge and non-edge cells are numbered.

(**f**,**h**,**j**,**l**) The intensity of the sGFP signals in each cell of excised leaves (**f**,**h**,**j**,**l**)of EF1αpro:sGFP-nosT #3 (**e**,**g**) and EF1αpro:sGFP-3'UTR #18 (**i**,**k**) (1 to 8 correspond to cells in [**e**], [**g**], [**i**] and [**k**], respectively). Red and black lines indicate the sGFP intensity in cells that were and were not reprogrammed into stem cells, respectively.

Scale bars represent 50 μm in (**e**), (**g**), (**i**), and (**k**).

Supplementary Figure 5

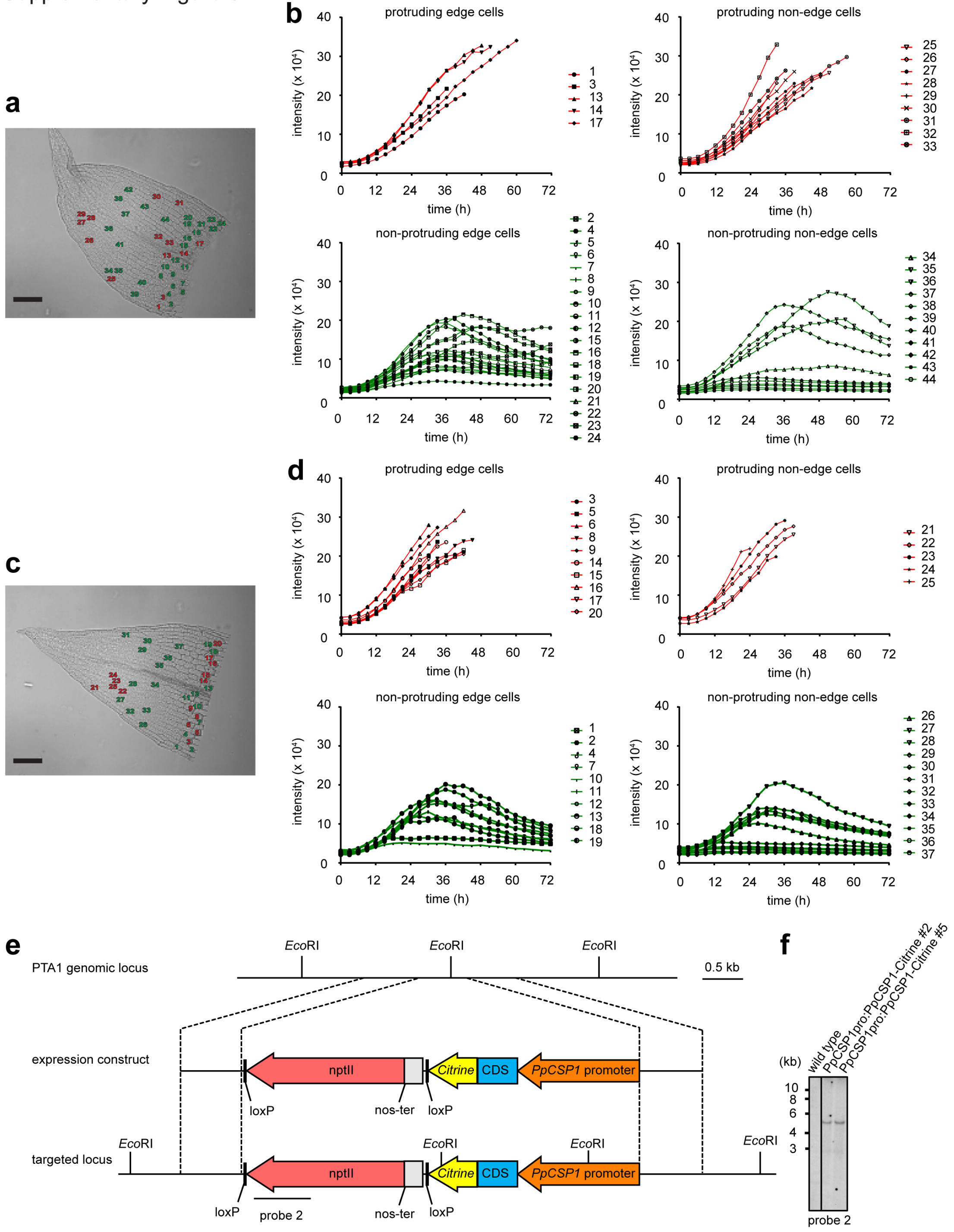

Supplementary Figure 5. Construction of the PpCSP1pro:PpCSP1-Citrine lines

(**a-d**) Replicates of quantitative analysis of Citrine signals of nPpCSP1-Citrine-nosT #136 line. Cut leaves of nPpCSP1-Citrine-nosT #136 line used for quantitative analysis of Citrine

signals. All edge cells and several non-edge cells are numbered for (**b**) and (**d**). The intensity of the Citrine signals in each cell (1 to 44 correspond to cells in [**a**]; 1 to 37 correspond to cells in [**c**], respectively) of excised leaves of nPpCSP1-Citrine-nosT #136. Red and green lines indicate intensities of Citrine signals in protruding and non-protruding cells, respectively.

(**e**) Schematic showing the insertion of *PpCSP1* promoter-driven *PpCSP1-Citrine* fusion gene construct into the PTA1 locus. Orange, blue, yellow, and pink arrows denote the *PpCSP1* promoter, the *PpCSP1* coding sequence (CDS), Citrine CDS, and the nptII expression cassette, respectively. Gray and black boxes denote the nosT and the loxP sequence, respectively. The probe used in (**f**) is indicated.

(**f**) DNA gel-blot analysis of targeted lines. Genomic DNA of wild type and PpCSP1pro:PpCSP1-Citrine (#2 and #5) lines was digested with *Eco*RI.

Scale bars represent 100 μm in (**a**) and (**c**).

Supplementary Figure 6

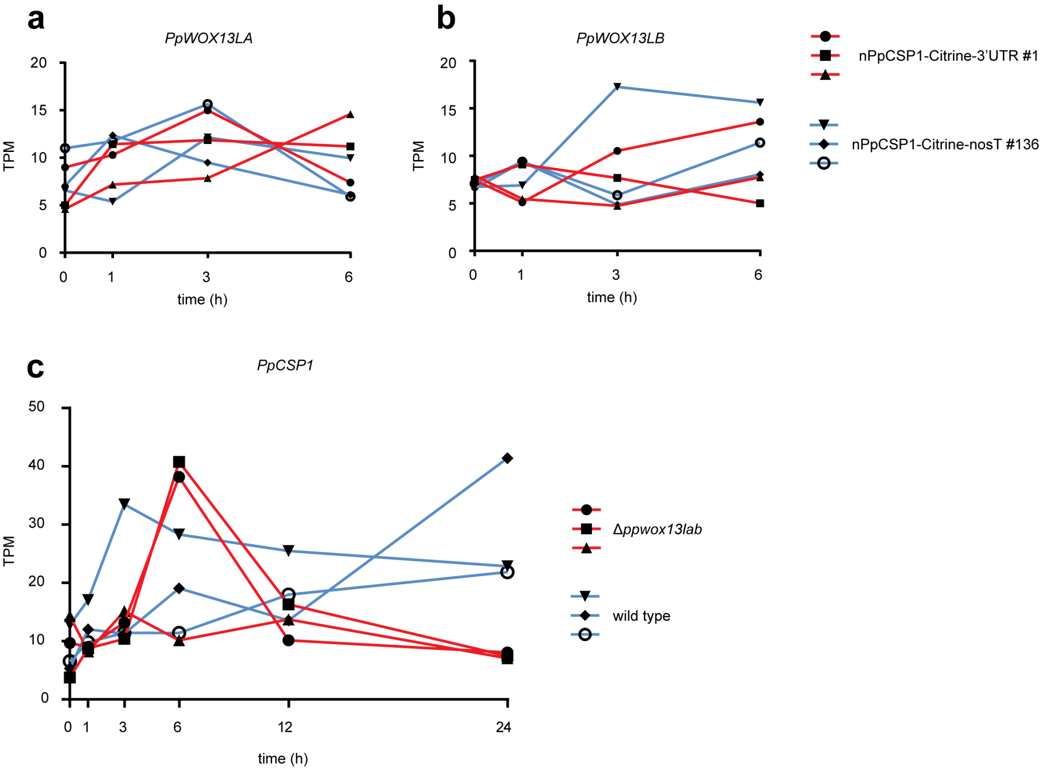

Supplementary Figure 6. Transcript levels of *PpWOX13L* genes in nPpCSP1-Citrine-3'UTR and nPpCSP1-Citrine-nosT lines and those of *PpCSP1* gene in the wild type and Δ*ppwox13lab* lines

(**a**,**b**) Transcript levels of *PpWOX13LA* and *PpWOX13LB* genes in nPpCSP1-Citrine-3'UTR and nPpCSP1-Citrine-nosT lines. Horizontal axes indicate the time after leaf excision and vertical axes indicate tags per million (TPM) values in 5'-DGE analysis. Red and blue lines indicate transcript levels in the nPpCSP1-Citrine-3'UTR and nPpCSP1-Citrine-nosT lines, respectively. Results of three independent experiments are shown.

(**c**) Transcript levels of *PpCSP1* gene in wild type and Δ*ppwox13lab* line. Horizontal axis indicates the time after leaf excision and vertical axis indicates tags per million (TPM) values in 5'-DGE analysis [11]. Blue and red lines indicate transcripts levels in the wild type and Δ*ppwox13lab* line, respectively. Results of three independent experiments are shown.

Supplementary Figure 7

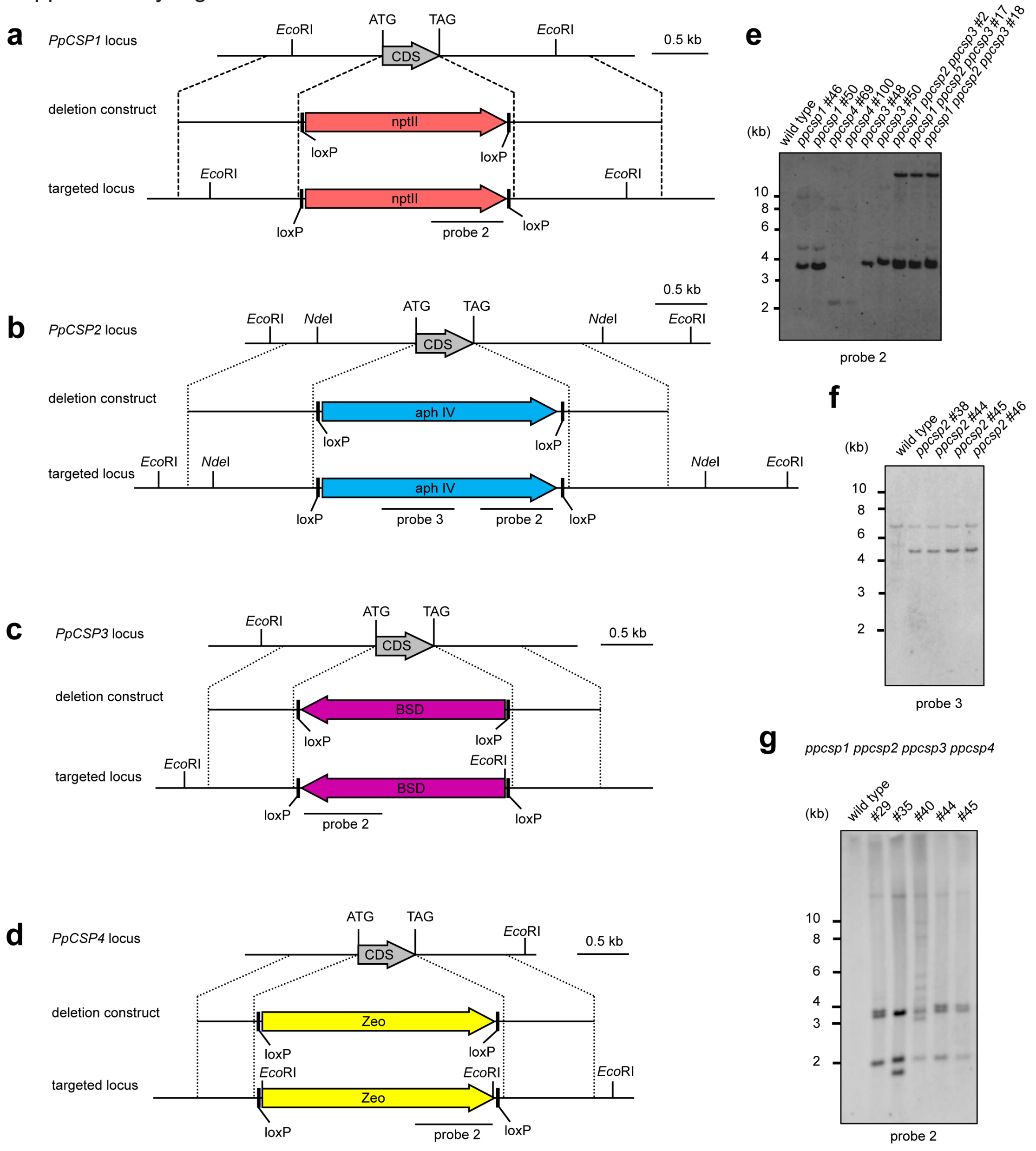

Supplementary Figure 7. Construction of the *PpCSP* deletion mutant

(**a**-**d**) Schematics of constructs targeting the *PpCSP1* (**a**), *PpCSP2* (**b**), *PpCSP3* (**c**), and *PpCSP4* (**d**) loci. Grey arrows represent the coding regions of *PpCSP1*, *PpCSP2*, *PpCSP3*, and *PpCSP4* in (**a**) to (**d**), respectively. Pink, blue, purple and yellow arrows indicate the neomycin phosphotransferase II expression cassette (nptII) [2], the aminoglycoside

phosphotransferase expression cassette (aph IV) [9,12], the blasticidin S deaminase expression cassette (BSD) [1], and Zeocin resistance cassette (Zeo) [9], respectively. Probes used in (**e**-**g**) are indicated. Procedures to make quadruple deletion mutants are described in Methods.

(**e**-**g**) DNA gel-blot analyses of targeted lines. Genomic DNA of wild type, *ppcsp1* (#46 and #50), *ppcsp4* (#69 and #100), *ppcsp3* (#48 and #50), and *ppcsp1 ppcsp2 ppcsp3* (#2, #17, and #18) lines was digested with *Eco*RI and hybridized with probe 2 (**e**). Genomic DNA of wild type and *ppcsp2* (#38, #44, #45 and #46) lines was digested with *Nde*I and hybridized with probe 3 (**f**). Genomic DNA of wild type and *ppcsp1 ppcsp2 ppcsp3 ppcsp4* (#29, #35, #44 and #45) lines was digested with *Eco*RI and hybridized with probe 2 (**g**).

Supplementary Figure 8

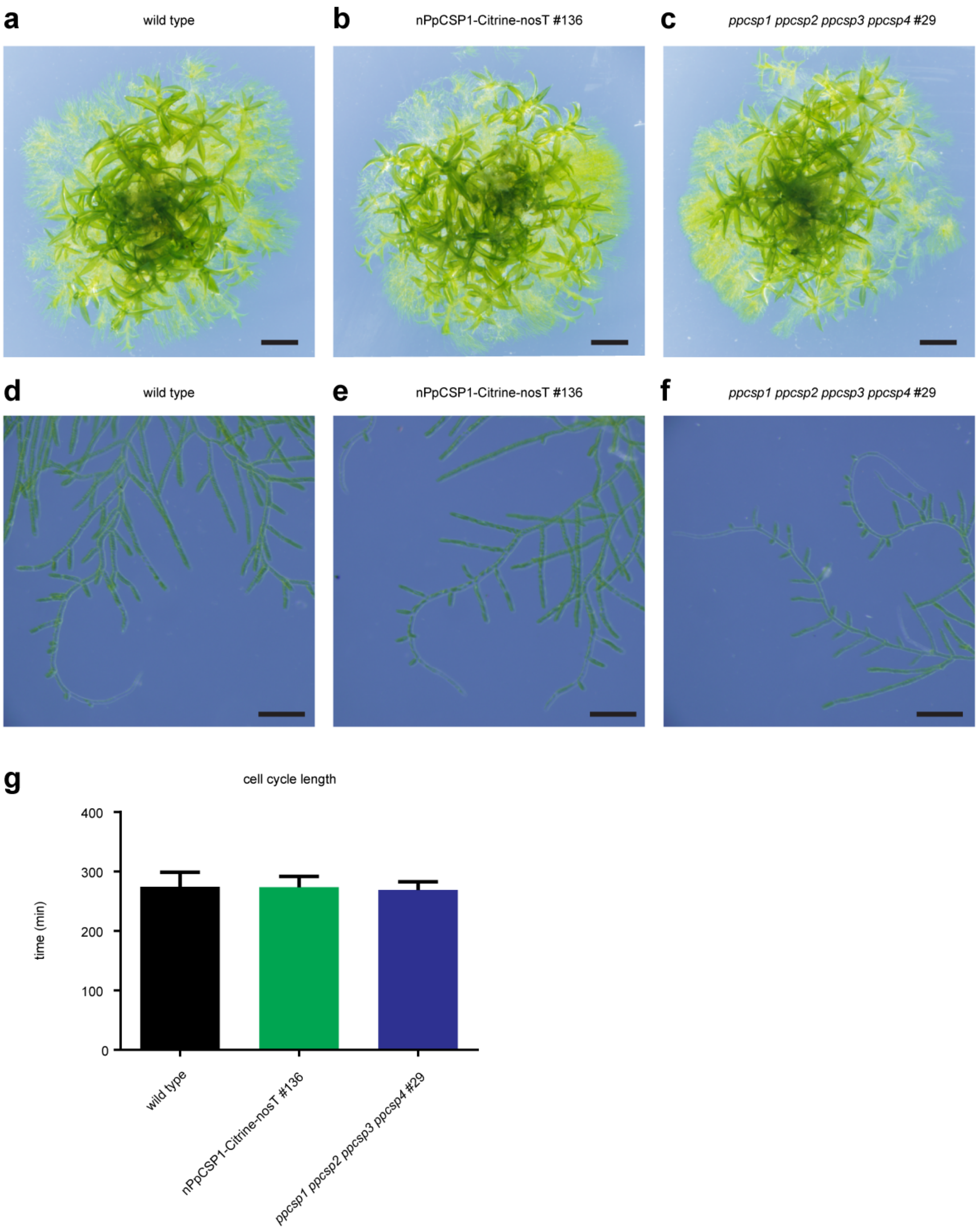

Supplementary Figure 8. Protonemata and gametophores of the wild type, nPpCSP1-Citrine-nosT line, and quadruple deletion mutant line

(**a-f**) Representative 4-week-old plants (**a-c**) and protonemata (**d-f**) of wild-type (**a,d**), nPpCSP1-Citrine-nosT #136 (**b,e**), and *ppcsp1 ppcsp2 ppcsp3 ppcsp4* #29 deletion mutant lines (**c,f**).

(**g**) The cell cycle duration of protonemata in the wild type, nPpCSP1-Citrine-nosT #136 line, and *ppcsp1 ppcsp2 ppcsp3 ppcsp4* #29 line. Error bars represent SD (n > 40). See also Supplementary Movie 6.

Scale bars represent 2 mm in (**a-c**) and 200 μm in (**d-f**).

Supplementary Figure 9

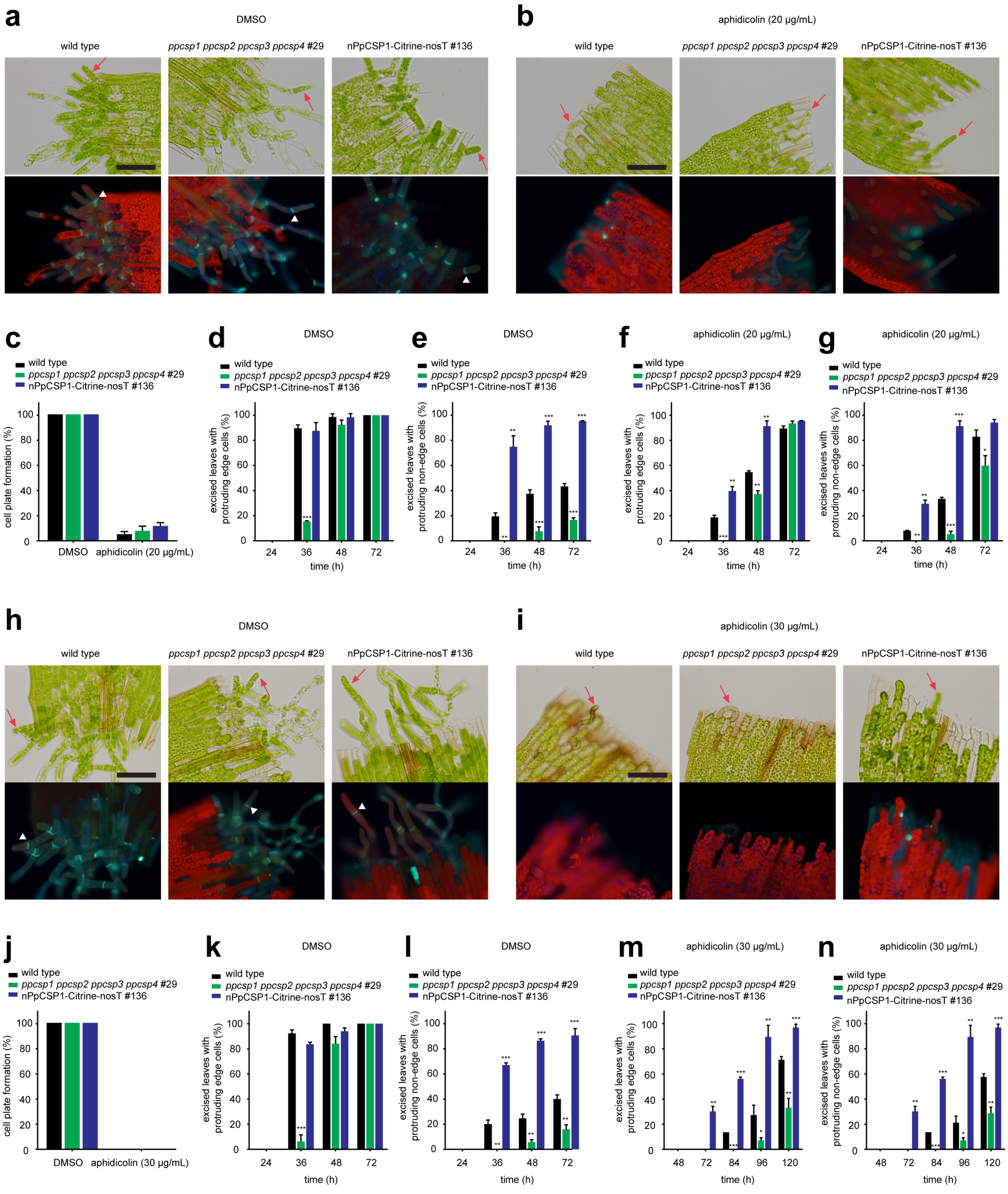

Supplementary Figure 9. Reprogramming and cell cycle progression with aphidicolin in the wild type, nPpCSP1-Citrine-nosT line, and quadruple deletion mutant line

(**a**,**b**) Bright-field and fluorescent images of excised leaves of the wild type, *ppcsp1 ppcsp2 ppcsp3 ppcsp4* #29 line, and nPpCSP1-Citrine-nosT #136 line incubated with or without 20

μg/mL aphidicolin for 72 h and stained with aniline blue to detect newly synthesized cell plates. Red arrows and white arrowheads indicate some cells with tip growth and newly synthesized cell plates, respectively.

(**c**) Percentage of excised leaves having at least one cell with cell plate formation (n = 20) after 72-h incubation with 20 μg/mL aphidicolin. Error bars represent SD from biological triplicates.

(**d**-**g**) Percentages of excised leaves with protruding edge cells (**d,f**) and protruding non-edge cells (**e,g**) without (**d,e**) or with (**f,g**) 20 μg/mL aphidicolin. Twenty leaves excised from the wild type, *ppcsp1 ppcsp2 ppcsp3 ppcsp4* #29 line, and nPpCSP1-Citrine-nosT #136 line were used for each analysis. Error bars represent SD from biological triplicates. *p < 0.05, ***p < 0.01, ***p < 0.001 by two-sided Welch's *t*-test.

(**h,i**) Bright-field and fluorescent images of excised leaves of the wild type, *ppcsp1 ppcsp2 ppcsp3 ppcsp4* #29 line, and nPpCSP1-Citrine-nosT #136 line incubated with or without 30 μg/mL aphidicolin for 72 h (DMSO) or 120 h (aphidicolin) and stained with aniline blue to detect newly synthesized cell plates. Red arrows and white arrowheads indicate some cells with tip growth and newly synthesized cell plates, respectively. For the control experiment with DMSO, we stopped the observation 72 h after the leaf excision, since chloronemata covered the excised leaves and further observation was impossible.

(**j**) Percentage of excised leaves having at least one cell with cell plate formation (n = 20) after 72-h incubation (DMSO) or 120-h incubation with 30 μg/mL aphidicolin. Error bars represent SD from biological triplicates.

(**k**-**n**) Percentages of excised leaves with protruding edge cells (**k,m**) and protruding non-edge cells (**l,n**) without (**k,l**) or with (**m,n**) 30 μg/mL aphidicolin. Twenty leaves excised from the wild type, *ppcsp1 ppcsp2 ppcsp3 ppcsp4* #29 line, and nPpCSP1-Citrine-nosT #136 line were used for each analysis. Error bars represent SD from biological triplicates. *p < 0.05, ***p < 0.01, ***p < 0.001 by two-sided Welch's *t*-test.

Scale bars represent 100 μm in (**a,b,h,i**).

Supplementary Table

Supplementary Table 1. Primer sequences used for RT-qPCR and plasmid construction

Primers used for RT-qPCR

Gene name		Sequence
PpCSP1	(F)	5'-GCCTGCGAGTCCGTCTTTC-3'
	(R)	5'-GGCGCAAAGATCCCAACA-3'
PpCSP2	(F)	5'-CTCCTGGTACTGTGAATAGTCGAGT-3'
	(R)	5'-GTCTCCTTCGCCTCCGTGCT-3'
PpCSP3,4	(F)	5'-CCGTCTTGCCTTTAGGTCCTTCTTT-3'
	(R)	5'-TCGCTCCCTCTTCCGCCATG-3'
sGFP	(F)	5'-GTCCGCCCTGAGCAAAGA-3'
	(R)	5'-TCCAGCAGGACCATGTGATC-3'
TUA1	(F)	5'-CGTAGGAGGGACCAGTTTGG-3'
	(R)	5'-TGCATTCATCCCCGAGTCA-3'

Primers used for probe amplification

Probe name		Sequence
Probe 1	(F)	5'-GCTGTCGGTCTTCGCCTGCG-3'
	(R)	5'-CCTCGACCTCCTCCATCGCC-3'
Probe 2	(F)	5'-TGAAATCACCAGTCTCTCTCTACAA-3'
	(R)	5'-GTTTTGATCTTGAAAGATCTTTTATCTTTAGA-3'
Probe 3	(F)	5'-ATATGAAGAAGCCTGAACTCA-3'
	(R)	5'-CTATTCCTTTGCCCTAGGACGAGTGCT-3'

Primers used for plasmid construction

Construct		Sequence
nPpCSP1-Citrine-nosT	5'(F)	5'-TCTCTCGAGAGCTGAGCTGGAATCGGG-3'
	5'(R)	5'-AGAATCGATGGAAGCGGCTGCAGGAGTG-3'
	3'(F)	5'-TCTACTAGTTAGGTGCTTCCGAGTAG-3'
	3'(R)	5'-AGAGCGGCCGCAAAAATCATCTACTCG-3'
pAK101	(F)	5'- GCATCTAGAGAGTTTTTGCAGGTAATCGAAGGTT -3'
	(R)	5'-GGGAAGCTTAAAAGCCTATACTGTACTTAACTTGATTG -3'
PpCSP1:LUC	(F)	5'-CACCAACGATTGTGCGACCATGCACACCGA-3'
	(R)	5'-CATAGCTGCTGCTGCGCCTCTCT-3'
EF1αpro:sGFP-nosT	(F)	5'-CACCATGGTGAGCAAGGGCGAGGAGCTGTTC-3'
	(R)	5'-TTACTTGTACAGCTCGTCCATGCC-3'
EF1αpro:sGFP-3'UTR	(F)	5'- CACCATGGTGAGCAAGGGCGAGGAGCTGTTC-3'
	(R)	5'-AAGGGCCCTCATCTACTCGAATGATTGCCTTCC-3'
3' UTR-623	(F)	5'-AAGGGCCCGTGCTTCCGAGTAGAAACAATTTTTCAAC-3'
	(R)	5'-AAGGGCCCTCATCTACTCGAATGATTGCCT TCC-3'
3' UTR-500	(R)	5'-AAGGGCCCTGACACAACATAAACCCTTCT CG-3'
3' UTR-400	(R)	5'-AAGGGCCCCATCCATCGCGGAGGAGCT-3'
3' UTR-300	(R)	5'-AAGGGCCCAAAGCAAACTTCTGCATACTACATTATAC C-3'
3' UTR-200	(R)	5'-AAGGGCCCAAAAAACAAAGTCAAGCAGAG CACTAC-3'
PpCSP1pro:PpCSP1-	(F)	5'-AATCCTCGAGCCCGGGAGCTTTGGATTCCATTGCACA C-3'
	(R)	5'- AATCTGTACACTAGGAAGCGGCTGCAGG-3'
ppcsp1	5'(F)	5'-TCTCTCGAGCATGTCTAAAGCTAACATCACC ACTG-3'
	5'(R)	5'-AGAATCGATAGCTGCTGCTGCGCCTCTCTG CCTTAC-3'
	3'(F)	5'-TCTCATATGGTGCTTCCGAGTAGAAACAATT TTTC-3'
	3'(R)	5'-AGAGGATCCCATCAATATACTCCTTGATGGC AGTC-3'
ppcsp2	5'(F)	5'-TCTCTCGAGGTGTAGTGCTGTTGTAATTCT CCTTG-3'
	5'(R)	5'-AGAATCGATAGCTCCCGACTCGACTATTCA CAGTAC-3'
	3'(F)	5'-TCTGGATCCTTGCTTCACTAGAGAGGCAAT GGTC-3'
	3'(R)	5'-AGAGAGCTCTGTATCCGTAAGGACGGATTA CTCTC-3'
ppcsp3	5'(F)	5'-AGGCTCGAGAAGCCAACGGTAAATCCCGA TAA-3'
	5'(R)	5'-CGCATCGATTGCTGCTGCGCCTCTCTGCG G-3'
	3'(F)	5'-TCTCCCGGGGCGTTTCCGAGTAGAAGC-3'
	3'(R)	5'-TCTCCGCGGCCACCTCTTCATCCCAATG-3'
ppcsp4	5'(F)	5'-TCTCCGCGGAATGGGTTCCCAACCAGC-3'
	5'(R)	5'-TCTCATATGTGCTGCTGCGCCTCTCTGC-3'
	3'(F)	5'-TCTATCGATGCGTTTCCGAGTAGAAGC-3'
	3'(R)	5'-TCTCTCGAGCCACCTCTTCATCCCAATG-3'

Supplementary References

1 Ishikawa, M. *et al.* Physcomitrella cyclin-dependent kinase A links cell cycle reactivation to other cellular changes during reprogramming of leaf cells. *Plant Cell* **23,** 2924-2938 (2011).

2 Nishiyama, T., Hiwatashi, Y., Sakakibara, I., Kato, M. & Hasebe, M. Tagged mutagenesis and gene-trap in the moss *Physcomitrella patens* by shuttle mutagenesis. *DNA Res.* **7,** 9-17 (2000).

3 Griesbeck, O., Baird, G. S., Campbell, R. E., Zacharias, D. A. & Tsien, R. Y. Reducing the environmental sensitivity of yellow fluorescent protein. Mechanism and applications. *J. Biol. Chem.* **276,** 29188-29194 (2001).

4 Kubo, M. *et al.* System for stable beta-estradiol-inducible gene expression in the moss *Physcomitrella patens*. *PLoS One* **8,** e77356 (2013).

5 Okano, Y. *et al.* A polycomb repressive complex 2 gene regulates apogamy and gives evolutionary insights into early land plant evolution. *Proc. Natl. Acad. Sci. USA* **106,** 16321-16326 (2009).

6 Odell, J., Caimi, P., Sauer, B. & Russell, S. Site-directed recombination in the genome of transgenic tobacco. *Mol. Genet. Genomics* **223,** 369-378 (1990).

7 Aoyama, T. *et al.* AP2-type transcription factors determine stem cell identity in the moss *Physcomitrella patens*. *Development* **139,** 3120-3129 (2012).

8 Chiu, W. *et al.* Engineered GFP as a vital reporter in plants. *Curr. Biol.* **6,** 325-330 (1996).

9 Hiwatashi, Y. *et al.* Kinesins are indispensable for interdigitation of phragmoplast microtubules in the moss *Physcomitrella patens*. *Plant Cell* **20,** 3094-3106 (2008).

10 Campbell, R. E. *et al.* A monomeric red fluorescent protein. *Proc. Natl. Acad. Sci.*

USA **99,** 7877-7882 (2002).

11 Sakakibara, K. *et al. WOX13-like* genes are required for reprogramming of leaf and protoplast cells into stem cells in the moss *Physcomitrella patens*. *Development* **141,** 1660-1670 (2014).

12 Sakakibara, K., Nishiyama, T., Deguchi, H. & Hasebe, M. Class 1 KNOX genes are not involved in shoot development in the moss *Physcomitrella patens* but do function in sporophyte development. *Evol. Dev.* **10,** 555-566 (2008).
